# The Economic Implications of the Covid-19 Pandemic on Older Adults in the U.S

**DOI:** 10.1093/geroni/igaf122.2336

**Published:** 2025-12-31

**Authors:** Michael Vetter

**Affiliations:** MGH - Center for Optimal Aging and Serious Illness, Danvers, Massachusetts, United States

## Abstract

Older adults were among those most disproportionately affected by the Covid-19 pandemic. Research over previous years has outlined the changes to health, loneliness, and long-term well-being outcomes, in addition to economic impacts. This project recognizes and addresses a gap in the literature pertaining to the role of government economic impact payments (EIP) and labor force participation during the pandemic for older adults. Leveraging the Health and Retirement Study’s 2020 Core Survey and 2021 “Perspectives on the Pandemic” supplement, results highlight the demographic characteristics of the population of respondents (n = 2167) and the distribution of changes to economic and labor outcomes resulting from the pandemic. Most respondents noted having received an EIP and used this payment to primarily pay off existing debt, followed closely by contributing to savings. The majority were not working at the time of receipt and saw few changes to their economic situation throughout the pandemic. However, a stratified analysis of the payment amount showed that women received, overall, 31% less than men. Findings indicate that while labor force participation among this group was low, use of the economic payment indicates a need to address personal debt. Moreover, the gendered difference in total amount received may be the result of several factors, including the division of labor and labor force participation in older generations and the responsibilities of caregiving duties falling to women rather than men over the life course.

